# Comparison of Medical School Financing Plans Among Matriculating US Medical Students From 2017 to 2019

**DOI:** 10.1001/jamanetworkopen.2021.17704

**Published:** 2021-07-20

**Authors:** Arman A. Shahriar, Varun Sagi, Lorenzo Adolfo Castañón-Gonzalez, Thomas E. Kottke, Gabriela Vazquez-Benitez, Renée Crichlow

**Affiliations:** 1 University of Minnesota Medical School, Minneapolis; 2 HealthPartners Institute, Minneapolis, Minnesota; 3 Codman Square Health Center, Boston, Massachusetts; 4 Department of Family Medicine, Boston University, Boston, Massachusetts

## Abstract

This survey study explores US students’ financing plans for medical school overall and by several demographic factors, focusing on race/ethnicity and household income.

## Introduction

Since the 1960s, the typical cost of a US medical education has outpaced inflation by 750% to reach approximately $300 000.^1^ This is concerning, given the compelling national interest in building a diverse physician workforce, because high costs create challenges for lower-income students, who are disproportionately from racial/ethnic groups underrepresented in medicine, such as individuals identifying as Black or Hispanic.^2,3^

Little is known about how students finance medical school, and with increasing costs, financing disparities could undermine equity in the path toward a more socioeconomically, racially, and ethnically diverse workforce. This study explores medical student financing plans overall and by several demographic factors, focusing on race/ethnicity and household income.

## Methods

This survey study was deemed not human participants research and therefore exempt from approval and informed consent by the University of Minnesota institutional review board. This study is reported following the American Association for Public Opinion Research (AAPOR) reporting guideline. Further details are provided in eMethods in the Supplement.

We analyzed deidentified, individual-level data from the 2017 to 2019 Association of American Medical Colleges (AAMC) Matriculating Student Questionnaire (MSQ; response rate, 65%-71%), in which students reported their financing plans using percentages totaling 100% (eMethods in the Supplement). We included students who answered questions assessing financing, household income, and plans to work in underserved areas, and compared characteristics of included and excluded individuals (eMethods in the Supplement). Financing responses were categorized as family or personal, loans, scholarship, or service, and their use was described overall and by demographic factors.

We defined a primary source of financing for a student as more than 50% of financing from a single category, and full-ride scholarship as 100% of financing from scholarship. Using multivariable logistic regression, we calculated adjusted odds ratios (aORs) with 95% Wald CIs for different primary sources and full-ride scholarships by self-identified race/ethnicity and household income group. Statistical analyses were conducted from December 24, 2020, to February 1, 2020, using SAS/STAT version 9.4 (SAS Institute).

## Results

Of all 44 903 respondents to the 2017 to 2019 MSQ, 29 725 (66.2%) were included. Approximately one-half of all respondents were in the highest household-income quintile (15 366 respondents [51.7%]) and 7233 respondents (24.3%) were in the top 5%. The largest racial/ethnic groups were non-Hispanic White, with 16 461 respondents (55.4%); non-Hispanic Asian, with 6330 respondents (21.3%); non-Hispanic Black, with 1931 respondents (6.5%); and Hispanic, with 3217 respondents (10.8%). Excluded respondent characteristics were similar apart from survey year. On aggregate, students expected a median (interquartile range) of 70% (10%-90%) of funds from loans, 5% (0%-30%) from family or personal, 1% (0%-20%) from scholarships, and 0% (0%-0%) from service (Table 1).

**Table 1.** Sample Characteristics and Descriptive Statistics of Medical School Financing Plans

Characteristic	Matriculants, No. (%)	Financing plan, %^a^							
		Family or personal		Loans		Scholarship		Service	
		Mean (SD)	Median (IQR)	Mean (SD)	Median (IQR)	Mean (SD)	Median (IQR)	Mean (SD)	Median (IQR)
All	29 725	22.06 (32.14)	5 (0-30)	56.57 (38.02)	70 (10-90)	16.01 (26.84)	1 (0-20)	4.61 (19.26)	0 (0-0)
MSQ year^b^									
2017	10 938 (36.8)	22.94 (32.73)	7 (0-30)	56.68 (38.01)	70 (15-90)	14.78 (25.67)	0 (0-20)	4.71 (19.33)	0 (0-0)
2018	9442 (31.8)	21.32 (31.52)	5 (0-25)	57.32 (37.60)	70 (20-90)	16.07 (26.68)	1 (0-20)	4.57 (19.28)	0 (0-0)
2019	9345 (31.4)	21.78 (32.04)	5 (0-30)	55.69 (38.43)	70 (10-90)	17.41 (28.22)	1 (0-20)	4.53 (19.18)	0 (0-0)
Race/ethnicity^c^									
Non-Hispanic									
White	16 461 (55.4)	21.35 (31.33)	5 (0-27)	59.84 (37.83)	75 (25-93)	12.66 (24.15)	0 (0-11)	5.37 (21.03)	0 (0-0)
Black	1931 (6.5)	6.63 (15.67)	0 (0-6)	56.67 (35.40)	66 (25-90)	31.51 (32.76)	20 (0-50)	4.64 (18.45)	0 (0-0)
Asian	6330 (21.3)	32.69 (36.81)	15 (0-60)	48.66 (38.48)	50 (0-85)	15.47 (26.56)	0 (0-20)	2.41 (13.52)	0 (0-0)
Hispanic (any race)	3217 (10.8)	13.63 (25.70)	0 (0-12)	57.72 (36.50)	70 (25-90)	23.54 (30.06)	10 (0-40)	4.48 (18.60)	0 (0-0)
American Indian or Alaska Native	233 (0.8)	11.78 (23.93)	0 (0-10)	48.52 (39.19)	50 (0-89)	25.79 (32.09)	10 (0-40)	13.44 (31.7)	0 (0-0)
Other or multiple	1553 (5.2)	24.44 (34.07)	6 (0-35)	52.81 (39.02)	65 (0-90)	17.44 (28.54)	1 (0-20)	4.40 (18.5)	0 (0-0)
Household income group^d^									
96%-100% (top 5%)	7233 (24.3)	43.21 (39.72)	30 (4-90)	42.12 (39.62)	40 (0-80)	11.01 (24.06)	0 (0-10)	2.96 (15.62)	0 (0-0)
81%-95%	8133 (27.4)	21.73 (30.25)	10 (0-30)	58.24 (37.49)	70 (20-90)	14.28 (25.97)	0 (0-15)	5.03 (20.34)	0 (0-0)
61%-80%	7112 (23.9)	14.77 (24.98)	5 (0-18)	62.95 (36.03)	77 (35-94)	16.17 (26.43)	3 (0-20)	5.42 (20.88)	0 (0-0)
41%-60%	3468 (11.7)	9.26 (18.76)	0 (0-10)	63.74 (34.92)	75 (40-94)	20.92 (28.47)	10 (0-30)	5.27 (20.42)	0 (0-0)
0%-40% (bottom 40%)	3779 (12.7)	7.73 (18.11)	0 (0-9)	62.06 (34.93)	75 (35-90)	24.53 (29.96)	10 (0-40)	4.73 (18.76)	0 (0-0)
Sex									
Men	14 135 (47.6)	21.36 (31.79)	5 (0-25)	56.45 (38.23)	70 (15-90)	16.35 (27.30)	1 (0-20)	5.07 (20.45)	0 (0-0)
Women	15 590 (52.4)	22.69 (32.45)	5 (0-30)	56.68 (37.83)	70 (20-90)	15.70 (26.40)	1 (0-20)	4.19 (18.11)	0 (0-0)
Age category, y									
<23	10 144 (34.1)	25.31 (33.87)	10 (0-40)	53.06 (38.77)	63 (0-90)	16.57 (27.41)	1 (0-20)	4.41 (19.16)	0 (0-0)
23-25	15 078 (50.7)	21.56 (31.81)	5 (0-27)	57.84 (37.57)	70 (20-90)	15.81 (26.83)	0 (0-20)	4.14 (18.19)	0 (0-0)
26-28	3053 (10.3)	17.59 (29.36)	2 (0-20)	61.09 (36.91)	75 (29-93)	15.17 (25.43)	1 (0-20)	5.09 (19.64)	0 (0-0)
>28	1450 (4.9)	13.94 (25.11)	0 (0-20)	58.40 (37.74)	70 (20-92)	16.03 (25.63)	2 (0-20)	9.94 (27.52)	0 (0-0)
School ownership									
Public	18 271 (61.5)	20.79 (31.18)	5 (0-25)	59.89 (37.79)	75 (25-92)	13.10 (23.96)	0 (0-15)	5.45 (21.03)	0 (0-0)
Private	11 454 (38.5)	24.07 (33.51)	8 (0-35)	51.27 (37.79)	59 (5-87)	20.66 (30.29)	5 (0-30)	3.27 (15.95)	0 (0-0)
Underserved area plans^e^									
Yes	9230 (31.1)	16.53 (27.85)	2 (0-20)	59.15 (36.43)	70 (25-90)	18.06 (27.18)	5 (0-25)	5.72 (20.47)	0 (0-0)
No	4801 (16.2)	26.66 (35.17)	10 (0-45)	50.94 (39.92)	60 (0-90)	16.66 (29.24)	0 (0-20)	4.72 (20.27)	0 (0-0)
Undecided	15 694 (52.8)	23.90 (33.08)	10 (0-34)	56.78 (38.16)	70 (15-90)	14.61 (25.76)	0 (0-18)	3.93 (18.15)	0 (0-0)

Abbreviations: IQR, interquartile range; MSQ, Matriculating Student Questionnaire.

^a^ Financing category definitions: family-personal includes the responses “money from parents, guardians, or other relatives,” “personal income and savings,” and “money earned by spouse or partner.” Each year, 77% to 78% of this category was “money from parents, guardians, or other relatives,” 18% to 19% was “personal income and savings,” and 3% to 4% was “money earned by spouse or partner.” Service includes the responses “scholarships or awards with a service commitment (NHSC, military, etc)” and “work-study program.” Each year, 91% to 93% was “scholarships or awards with a service commitment (NHSC, military, etc.)” and 7% to 9% was “work-study program.” Loans includes the response “loans.” Scholarship includes the response “scholarships or awards.”

^b^ Starting in 2018, respondents were given an option to enter “unknown”; therefore, fewer participants reported parental income starting in 2018.

^c^ Non-Hispanic Black includes individuals identifying as Black or African American, African, African American, Afro-Caribbean, and other Black or African American; non-Hispanic Asian includes Bangladeshi, Cambodian, Chinese, Filipino, Indian, Indonesian, Japanese, Korean, Laotian, Pakistani, Taiwanese, Vietnamese, and other Asian; Hispanic includes Hispanic, Latino, or Spanish origin, Argentinean, Colombian, Cuban, Dominican, Mexican, Mexican American, Chicano/Chicana, Peruvian, Puerto Rican, and other Hispanic, alone or combined with any racial identity; American Indian or Alaska Native includes American Indian and Alaska Native, alone or in combination; and other or multiple includes students identifying as more than 1 of the racial/ethnic categories, as well as Native Hawaiian, other Pacific Islander, Guamanian, Samoan, unknown, and other.

^d^ Census household income group percentile limits in current dollars: 40%: $45 600 (2017), $47 218 (2018), $50 000 (2019); 60%: $74 869 (2017), $77 150 (2018), $79 542 (2019); 80%: $121 018 (2017), $126 603 (2018), $130 000 (2019); 95%: $225 251 (2017), $244 088 (2018), $248 728 (2019). Limits from 2016, 2017, and 2018, as students were asked to estimate combined gross parental income for last year.

^e^ Questionnaire language: “Do you plan to work primarily in an underserved area?”

Most students (26 423 respondents [88.9%]) had a primary source of financing, with 17 328 respondents (58.3%) having loans as the primary financing source, 4717 respondents (15.9%) with a family or personal primary financing source, 3111 respondents (10.5%) with scholarships as their primary financing source, and 1267 respondents (4.3%) with service as the primary financing source (Table 2). Compared with White students, a family or personal primary financing source was less likely for Black students (aOR, 0.31; 95% CI, 0.24-0.42) but more likely for Asian students (aOR, 2.62; 95% CI, 2.42-2.84), and a primary scholarship source was more likely for both Black (aOR, 3.24; 95% CI, 2.85-3.68) and Hispanic (any race) students (aOR, 1.95; 95% CI, 1.73-2.19).

**Table 2.** Comparison of Different Primary Financing Sources and Full-Ride (100%) Scholarship by Race/Ethnicity and Household Income Group^a^

Group	Primary source								Full-ride scholarship^c^	
	Family-personal		Loans		Scholarship^b^		Service			
	No. (%)	aOR (95% CI)^d^	No. (%)	aOR (95% CI)^d^	No. (%)	aOR (95% CI)^d^	No. (%)	aOR (95% CI)^d^	No. (%)	aOR (95% CI)^d^
All	4717 (15.9)	NA	17 328 (58.3)	NA	3111 (10.5)	NA	1267 (4.3)	NA	1089 (3.7)	NA
Race/ethnicity										
Non-Hispanic										
White	2444 (14.8)	1 [Reference]	10 308 (62.6)	1 [Reference]	1234 (7.5)	1 [Reference]	840 (5.1)	1 [Reference]	537 (3.3)	1 [Reference]
Black	54 (2.8)	0.31 (0.24-0.42)	1100 (57)	0.62 (0.56-0.69)	477 (24.7)	3.24 (2.85-3.68)	77 (4)	0.73 (0.57-0.93)	115 (6)	2.07 (1.67-2.58)
Asian	1630 (25.8)	2.62 (2.42-2.84)	3083 (48.7)	0.54 (0.51-0.58)	635 (10)	1.14 (1.02-1.26)	128 (2)	0.41 (0.34-0.50)	230 (3.6)	1.01 (0.86-1.19)
Hispanic (any race)	281 (8.7)	0.87 (0.76-1.00)	1878 (58.4)	0.70 (0.65-0.76)	531 (16.5)	1.95 (1.73-2.19)	126 (3.9)	0.74 (0.61-0.91)	126 (3.9)	1.23 (1.00-1.50)
American Indian or Alaska Native	18 (7.7)	0.71 (0.43-1.18)	116 (49.8)	0.47 (0.36-0.61)	43 (18.5)	2.85 (2.03-4.02)	33 (14.2)	2.67 (1.83-3.92)	12 (5.2)	1.84 (1.02-3.33)
Other or multiple^e^	290 (18.7)	1.51 (1.30-1.74)	843 (54.3)	0.69 (0.62-0.77)	191 (12.3)	1.55 (1.31-1.83)	63 (4.1)	0.82 (0.63-1.07)	69 (4.4)	1.35 (1.04-1.75)
Household income group										
96%-100% (top 5%)	2671 (36.9)	1 [Reference]	3014 (41.7)	1 [Reference]	518 (7.2)	1 [Reference]	197 (2.7)	1 [Reference]	241 (3.3)	1 [Reference]
81%-95%	1163 (14.3)	0.26 (0.24-0.29)	4905 (60.3)	2.21 (2.07-2.36)	741 (9.1)	1.32 (1.17-1.48)	383 (4.7)	1.76 (1.48-2.10)	326 (4)	1.26 (1.06-1.50)
61%-80%	606 (8.5)	0.16 (0.14-0.17)	4681 (65.8)	2.77 (2.58-2.97)	716 (10.1)	1.48 (1.31-1.67)	362 (5.1)	1.81 (1.51-2.26)	239 (3.4)	1.09 (0.91-1.32)
41%-60%	143 (4.1)	0.08 (0.06-0.10)	2320 (66.9)	2.96 (2.71-3.23)	471 (13.6)	1.96 (1.71-2.25)	168 (4.8)	1.68 (1.36-2.08)	136 (3.9)	1.31 (1.05-1.63)
0%-40% (bottom 40%)	134 (3.5)	0.06 (0.05-0.08)	2408 (63.7)	2.75 (2.53-3.00)	665 (17.6)	2.49 (2.18-2.84)	157 (4.2)	1.45 (1.16-1.81)	147 (3.9)	1.31 (1.05-1.62)

Abbreviations: aOR, adjusted odds ratio; NA, not applicable.

^a^ Primary source was defined as more than 50% of total funds. Overall, 26 423 respondents (88.9%) of students entered a response of more than 50% within 1 category and were labeled as having a primary financing source within that category.

^b^ Primary scholarship is defined as more than 50% of funds projected from the scholarship category alone and by definition includes all students within the full-ride scholarship category.

^c^ Full-ride scholarship is defined as 100% of funds projected from the scholarship category alone.

^d^ aORs are adjusted for all other covariates. For example, race/ethnicity aORs are adjusted for household income group, year, sex, age category, school ownership, and underserved area plans.

^e^ Includes students identifying as more than 1 of the racial/ethnic categories, as well as Native Hawaiian, other Pacific Islander, Guamanian, Samoan, unknown, and other.

Among students in the top 5% household-income group, a median (interquartile range) of 30% (4%-90%) of all expected funds were family or personal. A primary family or personal source was far less likely in other income groups, especially the bottom 40% (aOR, 0.06; 95% CI, 0.05-0.08). Lower-income students had greater odds of both loans and scholarships as primary financing sources. Full-ride scholarships were uncommon overall (1089 respondents [3.7%]), and their odds were similar across income groups.

## Discussion

This survey study of matriculating US medical students found that from 2017 to 2019, loans were the largest expected financing plan, but scholarships and family or personal financing were substantial, together totaling approximately 38% of all funds, and varied considerably across groups. Family or personal funds were concentrated among higher-income students and White or Asian students. The paucity of such financing among Black students may reflect the widening wealth gap,^4^ rooted in structural racism, and could help explain why debt burden is currently highest for Black medical school graduates.^5^

Overrepresentation of students from high-income families in medical schools is well known,^6^ but to our knowledge, this study is the first to demonstrate the prominent role these families play in financing, particularly for high-income students from the top 5% of US households, who collectively projected slightly less than one-half of all funds as family or personal. Among lower-income students, the heavier reliance on loans suggests inadequacy of current scholarship amounts and/or allocation to offset stark family or personal deficits. Observations regarding full-ride scholarships likely represent a relatively small number of students enrolled in tuition-free schools and MD-PhD programs.^5^

 In the absence of cost control,^1^ disparate access to family-personal financing could further disadvantage low-income trainees, a group that will likely grow with an expanding body of students from racial/ethnic groups that are currently underrepresented in medicine.

This exploratory, observational study has several limitations. The self-reporting of financing and income could have introduced social desirability and self-protection biases because these are sensitive topics for many people. Furthermore, there were no AAMC data available on accuracy or reliability of estimates, and these parameters may vary across demographic groups. A nonresponse bias could be compromising generalizability, owing to the 65% to 71% MSQ response rates and unknown nonrespondent characteristics. Additionally, findings could be confounded by unmeasured factors, such as geography or financial obligations (eg, outstanding debt) at matriculation.

## Notes

Supplement. eMethods.
