# Supplementary material for: Comparison of Medical School Financing Plans Among Matriculating US Medical Students From 2017 to 2019
Source: JAMA Netw Open. 2021 Jul 20;4(7):e2117704. doi: 10.1001/jamanetworkopen.2021.17704 (PMC8293019; doi:10.1001/jamanetworkopen.2021.17704)
Supplement: Supplement. — eMethods. [file jamanetwopen-e2117704-s001.pdf]

Supplemental Online Content

Shahriar AA, Sagi V, Castañón-Gonzalez LA, Kottke TE, Vazquez-Benitez G, Crichlow R. Comparison of medical school financing plans among matriculating US medical students from 2017 to 2019. *JAMA Netw Open*. 2021;4(7):e2117704.
doi:10.1001/jamanetworkopen.2021.17704

eMethods.

This supplemental material has been provided by the authors to give readers additional information about their work.

© 2021 Shahriar AA et al. *JAMA Network Open.*

eMethods.

Between 2017 and 2019, 44,903 students responded to the Association of American Medical Colleges - Matriculating Student Questionnaire (AAMC-MSQ).[1] Survey responses are gathered after students have received detailed financial aid information from their school, so they know the total cost and the levels of institutional and federal financial aid available to them.[2] For our study, we first excluded those who did not report on financing plans (n=6,125), followed by those who did not report parental income (additional n=8,939) and underserved area plans (additional n=114), bringing the excluded total to 15,178 and leaving 29,725 unique respondents for our study.

We were unable to assess MSQ non-respondent characteristics as these data were unavailable. Comparison between those included and excluded in our study was done using standardized differences, considering a threshold of < 0.20 (20%) as similar groups (**eTable**). We expected to exclude a larger percentage of 2018 and 2019 respondents, because beginning in 2018, respondents were given an option to enter "unknown" for parental income, resulting in fewer responses.[1]

Our definition of *primary source* of financing as >50% from one category was chosen as over half of funds expected from one category would imply that category as the leading expected source of financing for an individual, and the creation of these binary variables allowed for meaningful statistical comparisons. We additionally chose to report on "*full-ride scholarships"* as readers may be interested in the prevalence and distribution of these awards given the colloquial use of the phrase to refer to scholarships covering all of tuition and costs.

Regression models included year, self-identified race/ethnicity, household-income group (divided as top 5%, 81-95%, 61-80%, 41-60%, and bottom 40%), sex (male or female), and age

© 2021 Shahriar AA et al. *JAMA Network Open.*

category (<23, 23-25, 26-28, and >28). Additionally, we included school ownership (public or private) because private medical schools on aggregate have higher cost of attendance,[2] and plans to work in an underserved area (yes, no, or undecided) because these plans may theoretically influence financing methods such as service commitments.[2] Race/ethnicity was self-identified from a broad range of investigator-defined options. Race/ethnicity categories were heterogeneous (i.e., Asian, non-Hispanic), and their details are included in the footnotes of **Table 1**. Household-income groups were assigned using US Census income limits, and specific dollar thresholds are also provided in the footnotes of **Table 1**.[3] Statistical analyses were conducted from December 24, 2020, to February 1, 2020, using SAS/STAT 9.4 version (SAS Institute, Cary, NC).

American Association for Public Opinion Research (AAPOR) Standard Disclosure Checklist:

Basic Disclosure Element	Details
Survey sponsor	Association of American Medical Colleges
Survey/Data collection supplier	Association of American Medical Colleges
Population represented	65-71% of matriculating U.S. medical students
Sample size	MSQ sample size 2017-2019: 44,903<br>Study sample size: 29,725
Mode of data collection	Electronic survey; email
Type of sample	Census of all U.S. LCME-accredited medical schools
Start and end dates of data collection	MSQ 2017: 6/1/2017 - 9/15/2017<br>MSQ 2018: 6/1/2018 - 9/15/2018<br>MSQ 2019: 6/1/2019 - 9/15/2019
Margin of sampling error for total sample	Not applicable
Margin of sampling error for key subgroups	Not applicable
Are the data weighted?	Not applicable
Contact for more information	**Tyler Litsch, MPH, CHES**<br>Research and Data Analyst<br>Association of American Medical Colleges<br>tlitsch@aamc.org

© 2021 Shahriar AA et al. *JAMA Network Open.*

eReferences:

1. Association of American Medical Colleges. Matriculating student questionnaire. 2017-2019. Accessed Jan 26, 2021. https://www.aamc.org/data-reports/students-residents/report/matriculating-student-questionnaire-msq
2. Youngclaus J, Fresne JA. Physician Education Debt and the Cost to Attend Medical School: 2020 Update. Washington, DC. Association of American Medical Colleges, 2020.
3. U.S. Census Bureau, Current Population Survey, Annual Social and Economic Supplements (CPS ASEC). Accessed Feb 10, 2021. https://www.census.gov/data/tables/time-series/demo/income-poverty/historical-income-households.html.

© 2021 Shahriar AA et al. *JAMA Network Open.*

eTable. Characteristics of Included and Excluded Respondents, MSQ 2017-2019

	**Total N (%)**	**Included N (%)**	**Excluded N (%)[1]**	**Standardized Difference**
All	44903 (100.0)	29725 (100.0)	15178 (100.0)	
MSQ year				
2017	13868 (30.9)	10938 (36.8)	2930 (19.3)	0.38
2018	15447 (34.4)	9442 (31.8)	6005 (39.6)	0.16
2019	15588 (34.7)	9345 (31.4)	6243 (41.1)	0.20
Race/ethnicity				
White, non-Hispanic	24090 (53.6)	16461 (55.4)	7629 (50.3)	0.10
Black, non-Hispanic	3142 (7.0)	1931 (6.5)	1211 (8.0)	0.06
Asian, non-Hispanic	9953 (22.2)	6330 (21.3)	3623 (23.9)	0.06
Hispanic	4840 (10.8)	3217 (10.8)	1623 (10.7)	0.00
Am. Ind./Alaska Nat.	348 (0.8)	233 (0.8)	115 (0.8)	0.00
Other, multiple	2530 (5.6)	1553 (5.2)	977 (6.4)	0.05
Sex				
Male	20862 (46.5)	14135 (47.6)	6727 (44.3)	0.07
Female	24021 (53.5)	15590 (52.4)	8431 (55.5)	0.06
"Z"	20 (0.0)	0 (0.0)	20 (0.1)	NA
Age category, y				
< 23	15065 (33.6)	10144 (34.1)	4921 (32.4)	0.04
23-25	22535 (50.2)	15078 (50.7)	7457 (49.1)	0.03
26-28	4806 (10.7)	3053 (10.3)	1753 (11.5)	0.04
> 28	2497 (5.6)	1450 (4.9)	1047 (6.9)	0.09
School ownership				
Public	27346 (60.9)	18271 (61.5)	9075 (59.8)	0.03
Private	17557 (39.1)	11454 (38.5)	6103 (40.2)	0.03

[1] Of all 15178 excluded respondents, 6125 (40.4%) were missing financing plans, 14167 (93.3%) were missing household income and 3577 (23.6%) were missing undeserved plans.

© 2021 Shahriar AA et al. *JAMA Network Open.*

eAppendix. Questions Included for Analysis from the AAMC MSQ 2017-2019

The following questions from the AAMC Matriculating Student Questionnaire (MSQ) were included in this study. The questions are listed and numbered in the order in which they appear in the MSQ. All other questions are omitted from this Appendix. *indicates that the item is populated from other AAMC data sources (e.g., American Medical College Application Service [AMCAS])

1. Sex*
- Male
- Female

2. Age at matriculation*
- Under 20
- 20 through 22
- 23 through 25
- 26 through 28
- Over 28

3. How do you self-identify?* *Multiple responses allowed*
- American Indian or Alaska Native
- Asian
- Black or African American
- Hispanic, Latino, or Spanish origin
- Native Hawaiian or Other Pacific Islander
- White
- Other
- Non-U.S. citizen and non-permanent resident

4. Do you plan to WORK primarily in an underserved area?
- Yes
- No
- Undecided

5. Regardless of your dependency status, please indicate your parents' combined gross income for last year (a rough estimate is sufficient). Note: starting in 2018, an additional response option of unknown parental income was added. Therefore, fewer participants reported parental income starting in 2018.
- Free text

6. How do you plan to finance your medical school education? Please enter a percentage for each applicable category to total 100%.
- Scholarships or awards
- Scholarships or awards with a service commitment (NHSC, military, etc.)
- Loans
- Work-study program
- Personal income and savings
- Money from parents, guardians, or other relatives
- Money earned by spouse or partner
- Other

7. Control of medical school*
- Private
- Public

© 2021 Shahriar AA et al. *JAMA Network Open.*
